# Building QSAR for HTS in vitro assays - a study for the prediction of Aryl hydrocarbon receptor activators

**DOI:** 10.1186/1758-2946-5-S1-P51

**Published:** 2013-03-22

**Authors:** Ahmed M Abdelaziz, Alexander Safanyaev, Vladimir Palyulin, Igor V Tetko

**Affiliations:** 1 Institute of Structural Biology, Helmholtz Zentrum Muenchen, Neuherberg, Germany; 2 eADMET GmbH, Neuherberg, Germany; 3 Department of Chemistry, Moscow State University, Moscow, Russian Federation

## 

The Aryl hydrocarbon receptor (AHR) is a ligand-dependent transcription factor that, normally inactive, upon activation responds to exogenous and endogenous chemicals with the induction/repression of expression of large battery of genes. This induces diverse biological and toxic effects in a wide range of tissue. To evaluate the structural characteristics of small molecules responsible for AHR binding and toxicity, cross-validated qualitative QSAR models were developed using the free tool OCHEM (http://ochem.eu) by ASNN, random forests, J48 and MLR analyses and applicability domain was estimated. For the subset of most confident predictions the achieved accuracy can reach as high as 95%. Fragmental analysis of the chemical structures was applied to better understand the origin of AHR binding affinity. Analysis confirms several chemical scaffolds of halogenated aromatic hydrocarbons (HAHs) and polycyclic aromatic hydrocarbons (PAHs) as activators of the AHR. The high accuracy of in silico techniques in modeling the in vitro assay results endorses QSAR as a promising technique in predictive toxicology.

